# Trends in Meningococcal Disease in the United States Military, 1971–2010

**DOI:** 10.3201/eid1809.120257

**Published:** 2012-09

**Authors:** Michael P. Broderick, Dennis J. Faix, Christian J. Hansen, Patrick J. Blair

**Affiliations:** Naval Health Research Center, San Diego, California, USA

**Keywords:** Neisseria meningitidis, meningococcal disease, infectious disease epidemiology, vaccines, military personnel, bacteria, United States, serogroups, vaccine effectiveness, conjugate, polysaccharide, meningococci, *Suggested citation for this article*: Broderick MP, Faix DJ, Hansen CJ, Blair PJ. Trends in meningococcal disease in the United States military, 1971–2010. Emerg Infect Dis [serial on the internet]. 2012 Sep [*date cited*]. http://dx.doi.org/10.3201/eid1809.120257

## Abstract

Meningococci have historically caused extensive illness among members of the United States military. Three successive meningococcal vaccine types were used from 1971 through 2010; overall disease incidence dropped by >90% during this period. During 2006–2010, disease incidence of 0.38 (cases per 100,000 person-years) among members of the US military was not significantly different from the incidence of 0.26 among the age-matched US general population. Of the 26 cases in the US military, 5 were fatal, 15 were vaccine failures (e.g., illness in a person who had been vaccinated), and 9 were caused by *Neisseria meningitidis* serogroup Y. Incidences among 17- to 19-year-old basic trainees and among US Marines were significantly higher than among comparison military populations (p<0.05). No apparent change in epidemiology of meningococcal disease was observed after replacement of quadrivalent polysaccharide vaccine with conjugate vaccine in 2007. The data demonstrate that vaccination with meningococcal vaccine is effective.

## Abstract 2

When you consider the risks undertaken by US military personnel, do you include risk for disease? Public health officials do. Military personnel are at risk for infectious disease because of crowding, the rigors of physical training, and sometimes unhygienic field conditions. Meningococcal disease (usually manifested as bacterial meningitis or blood-borne infection) can be rapidly fatal. It has historically affected the military more than the general US population. One hundred years' worth of data support this trend from as long ago as World War I. However, in 1970, a policy requiring vaccination of military recruits started lowering the rate of infection, although the rate remained higher than that for the general population. Since 1982, improvements in vaccines have lowered rates even further. As a result of these vaccination efforts, the meningococcal disease rate among military personnel has reached a historic low, which now matches that of the general population.

Cases of meningococcal disease have long plagued the United States military, with incidences (defined as cases per 100,000 person-years) as high as 150 during World War I (*1*) and 80 during World War II (*2*). Corresponding incidences among the US general population were 9 and 16 during World War I and World War II, respectively (*2*). In 1969, the incidence among US Army basic trainees was 81 (*2*). Such elevated incidences relative to the non–age-matched US population were attributed to crowding and unhygienic conditions in unique environments. In particular, these factors imposed higher incidences of disease at military basic training centers. Contributing factors likely included the convergence of people from a wide geographic area and the extreme physical demands of basic military training.

During the past century, US military incidence of meningococcal disease has markedly decreased to converge with that of the (non–age-adjusted) US general population (Figure 1). Since the 1970s, the military has maintained a policy of universal meningococcal vaccination for all persons entering all branches of service. After the US Army’s early 1970s introduction to all incoming personnel of a vaccine targeting *N. meningitidis* serogroup C, disease rates dropped by >90% (*1*,*4*,*5*). However, during 1971–1989, the Army’s mean annual incidence remained significantly higher than that among the non–age-matched general population (3.6 vs. 1.02; p<0.0001; general population data from [*3*]).

**Figure 1:**
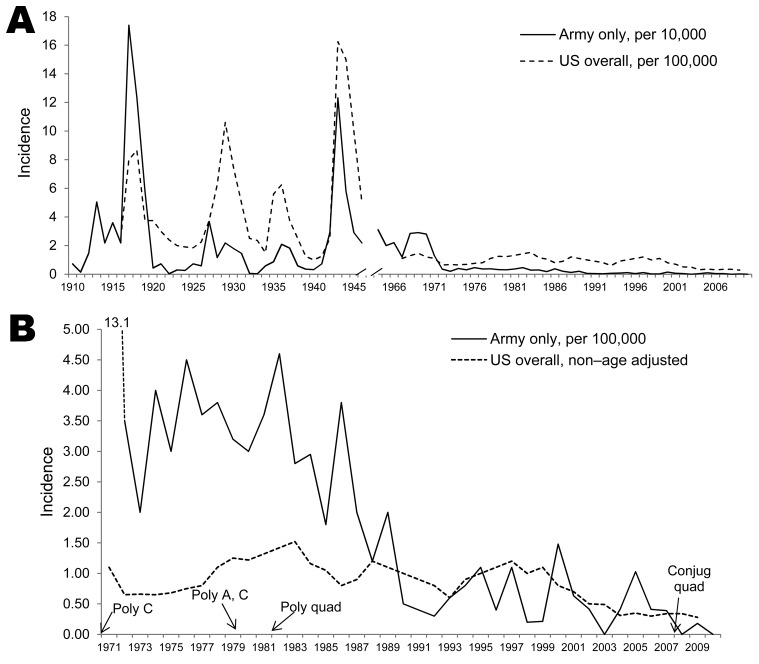
Timeline showing 100 years of meningococcal disease incidence in the US population compared with members of the US Army (A) and effects of introduction of meningococcal vaccines (B; years in which the vaccine types were introduced into the military indicated by arrows). Rates are unadjusted for age matching. Data for the US Army and the general population for 1910–1946 from Brundage and Zollinger (*2*). General population data for 1967–1977 from Brundage and Zollinger (*2*) and for 1978–2009 from the Centers for Disease Control and Prevention (*3*). US Army data for 1964–1998 from Brundage et al. (*1*), 1999–2010 from personal communication with the Armed Forces Health Surveillance Center, and 2006–2009 from Naval Health Research Center Meningococcal Disease Surveillance. The incidence in the Army from 1982–1989 of 2.1 cases per 100,000 person-years was deduced from Figure 1 in Brundage et al. (*1*) by using the percentage of each military division’s percentage contribution to the total active-duty military populations during 1982–1989, with each year’s percentages estimated from those of the 1990–1992 average. These percentages were stable within ±2 percentage points during 1990–2006. Poly, polysaccharide; C, *Neisseria meningitidis* serogroup C; A, *N. meningitidis* serogroup A; quad, quadravalent (*N. meningitidis* serogroups A, C, W-135, and Y); conjug, conjugate.

In 1982, a quadrivalent polysaccharide vaccine (MPSV-4; Menomune, Sanofi Pasteur, Bridgewater, NJ, USA) was introduced; this vaccine targets serogroups A, C, W-135, and Y. No broad-coverage vaccine against serogroup B exists (*6*). During 1982–1989, meningococcal disease incidence among members of the military was 2.1 (*1*); for 1990–2009, rates among both the Army and the US general population dropped significantly, with Army rates not significantly different from those observed in the general population (0.5 vs. 0.7; p = 0.19; general population data from [*5*]).

Despite declining incidence during the past 4 decades, the elevated susceptibility to meningococcal disease among members of the US military makes this population of interest regarding the performance of current vaccines. Of particular interest is the performance of the newer conjugate vaccine, MCV-4 (Menactra; Sanofi Pasteur, Swiftwater, PA, USA), which gradually replaced MPSV-4 in the military during 2006–2008. During 2009–2010, virtually all vaccinations were with MCV-4.

We report the epidemiology of 26 cases of meningococcal disease that occurred in members of the US military during 2006–2010. Demographics, geographic location, clinical syndrome, vaccination, and death rates are reviewed. Historical and current trends in the military are evaluated and compared with those of the US general population.

## Materials and Methods

### Case Information Collection

The Naval Health Research Center (NHRC) monitors reports from the Armed Forces Health Surveillance Center (AFHSC) Defense Medical Surveillance System database (*7*) and from the EpiData Center at the Navy and Marine Corps Public Health Center. Possible cases of meningococcal disease are identified from the Defense Medical Surveillance System database by using diagnostic code 036 from the International Classification of Diseases, 9th Revision, or by reports from military and US civilian laboratories that have identified *N. meningitidis* infection in active-duty military members. Possible cases without a confirmatory laboratory diagnosis are defined as probable by the presence of the appropriate clinical syndrome, with laboratory identification of encapsulated, gram-negative diplococci that are not identified as *N. meningitidis*. A case is defined as confirmed if the patient has the appropriate clinical syndrome and a laboratory identification of *N. meningitidis* is made by either PCR or culture. PCR and direct fluorescent antibody testing were used in our laboratories to identify a specific serogroup. The NHRC obtains case information and a variety of patient specimen types from the treating hospital.

### Statistical Analyses

Analysis of variance and Student *t* tests were used to compare incidences for which we did not have numerators or denominators over different periods within the Army and military populations and between the Army and military and the general population. The normal approximation was used for the comparison of incidences of >100 cases/year; for incidences <100 cases/year, Poisson modeling was used to evaluate differences between population strata (*8*). Demographic categories evaluated were patient age, sex, military rank, military branch, region, and military versus US general population; *N. meningitidis* serogroups were also compared.

## Results

### Cases

Of the 84 possible cases of meningococcal disease within US military that were reviewed during 2006–2010, the NHRC confirmed 23 cases by culture, PCR, or both; 1 case was determined to be probable. The NHRC also confirmed 2 cases that had not previously been identified by the AFHSC. The result of the surveillance was 25 confirmed cases and 1 probable case, of which 5 were fatal (Table 1); *N. meningitidis* serogroup breakdown was 6 B, 7 C, and 10 Y. The large number of possible cases reported among the military that were not confirmed were likely a result of miscoding of an initial or subsequent diagnosis; these included miscoding of a patient with viral or aseptic meningitis (n = 34), a final negative laboratory result after an initial diagnosis of meningococcal disease (n = 19), and a positive result on a nonsterile site only (n = 4).

**Table 1:** Confirmed and probable cases of meningococcal disease among members of the United States military, 2006–2010*

Case no.	Year	Q	Death	Clinical syndromes	Military branch	Patient age group, y/sex	Patient race/ ethnicity	Patient military status	Vaccine type	Months from vaccination to illness	*Neisseria meningitidis* serogroup
1	2006	1	Yes	Men, sep	A	20–24/M	B	AD	MPSV-4	12	C
2	2006	1	No	Men	M	17–19/M	W	AD	MPSV-4	7	C
3	2006	3	No	Men	M	20–24/M	W	Trainee	MPSV-4	NA	UNK
4	2006	3	No	Men, sep	A	17–19/M	W	Trainee	MPSV-4	1	C
5	2006	3	No	Men	M	17–19/M	W	Trainee	NA	1	UNK
6	2006	4	No	Men	M	20–24/M	W	AD	MPSV-4	7	C
7	2007	3	No	Men	N	25–29/M	W	AD	NA	NA	UNK
8	2007	3	Yes	Sep	A	25–29/M	W	AD	MPSV-4	62	B
9	2007	3	No	Sep	CG	35–39/M	UNK	AD	MPSV-4	229	B
10	2007	3	Yes	Sep	A	17–19/M	W	AD	MCV-4	3	C
11	2007	4	No	Sep	N	17–19/M	B	AD	NA	NA	Y
12	2008	1	No	Sep	M	17–19/M	B	Trainee	MPSV-4	3	Y
13	2008	1	No	Men	M	17–19/M	W	AD	MPSV-4	5	Y
14	2008	1	No	Men	M	17–19/M	W	Trainee	MCV-4	2	B
15	2008	2	No	Men	M	17–19/M	W	AD	MCV-4	9	UNK
16	2008	2	No	Men, sep	M	17–19/M	W	Trainee	MCV-4	1	B
17†	2008	3	Yes	Sep	AF	35–39/F	A	AD	MPSV-4	161	B
18	2008	4	No	Sep	M	20–24/M	H	Trainee	MCV-4	1	Y
19	2008	4	Yes	Men, sep	N	20–24/M	W	Cadet	MPSV-4	15	Y
20	2009	1	No	Men, sep	N	20–24/M	W	AD	MCV-4	4	Y
21	2009	1	No	Men	A	20–24/F	UNK	AD	MCV-4	7	B
22	2010	1	No	Sep	M	20–24/M	B	Trainee	MCV-4	1	Y
23	2010	2	No	Men, sep	N	20–24/F	W	AD	MPSV-4	43	Y
24	2010	3	No	Men, sep	M	17–19/M	W	Trainee	MCV-4	3	Y
25	2010	3	No	Men	M	25–29/F	B	AD	MCV-4	8	C
26	2010	4	No	Men	AF	20–24/M	B	AD	MCV-4	7	C

*Q, quarter; men, meningitis; sep, sepsis; A, Army; B, black; AD, active duty; MPSV-4, polysaccharide; M, Marine Corps; W, white; NA, not available (highly unlikely that person was not vaccinated, but vaccination cannot be confirmed); UNK, unknown; CG, Coast Guard; MCV-4, conjugate; N, Navy; AF, Air Force; A, Asian; H, Hispanic/Latino. †Probable case.

### Demographic and Clinical Variables

Relationships and covariations within demographics, military branch, military rank, geographic location, diagnosis, *N. meningitidis* serogroup, vaccination status, and fatalities were examined. Significant differences were found between stratifications within age (p<0.05), military branch (p<0.05), and rank (p<0.05) (Table 2).

**Table 2:** Cases of meningococcal disease (n = 26), by *Neisseria meningitidis* serogroup, within demographic and clinical categories, United States military, 2006–2010*

Characteristic	*N. meningitidis* serogroup, no. (%) cases					% Cases	% Total population	Incidence
	B	C	Y	Unknown	Total			
Total	6 (23)	7 (27)	9 (35)	4 (15)	26			
Year of illness								
2006	0	4	0	2	6	23	20	0.440
2007	2	1	1	1	5	19	20	0.367
2008	3	0	4	1	8	31	20	0.581
2009	1	0	1	0	2	8	20	0.142
2010	0	2	3	0	5	19	20	0.354
Death								
Yes	2	2	1	0	5	19		
No	4	5	8	4	21	81		
Age range, y								
17–19	2	3	4	2	11	42	7	**2.343**
20–24	1	3	5	1	10	38	33	0.434
25–29	1	1	0	1	3	12	23	0.188
30–34	0	0	0	0	0	0	14	0.000
35–39	2	0	0	0	2	8	12	0.250
>40	0	0	0	0	0	0	10	0.000
Clinical syndrome								
Meningitis alone	2	4	1	4	11	42		
Sepsis alone	3	1	4	0	8	31		
Both	1	2	4	0	7	27		
Vaccine type								
Conjugate	3	2	4	1	10	38		
Polysaccharide	3	4	4	2	13	50		
Unknown	0	1	1	1	3	12		
Years from vaccination to illness								
<1	NA	6	7	0	13	81		
1–2	NA	1	1	0	2	13		
2–3	NA	0	0	0	0	0		
>3	NA	0	1	0	1	6		
Military branch								
Air Force	1	1	0	0	2	8	23	0.120
Army	2	3	0	0	5	19	37	0.189
Coast Guard	1	0	0	0	1	4	3	0.509
Marine Corps	2	3	5	3	13	50	14	**1.353**
Navy	0	0	4	1	5	19	23	0.302
Location								
Continental United States	4	5	8	3	20	77	71	0.418
Outside continental United States	2	2	1	1	6	23	29	0.310
Race/ethnicity								
Asian	1	0	0	0	1	4	4	0.328
Black	0	3	3	0	6	23	18	0.472
Hispanic/Latino	0	0	1	0	1	4	12	0.123
White	3	4	5	4	16	62	61	0.367
Native American/Alaskan	0	0	0	0	0	0	2	0.000
Unknown	2	0	0	0	2	8	3	
Sex								
F	2	1	1	0	4	15	14	0.401
M	4	6	8	4	22	85	86	0.359
Military rank								
Recruit	2	1	4	2	9	35	12	**1.057**
Enlisted	4	6	5	2	17	65	73	0.330
Officer	0	0	0	0	0	0	15	**0.000**

*Exact denominators are not available in all categories. Percentage values may not equal 100 because of rounding. **Boldface** indicates that incidence for the category is significantly different from the incidence in the remaining population (Poisson p<0.05). NA, not applicable (no serogroup B vaccine).

### Age

During 2006–2010, incidence of meningococcal disease in the military among those >17 years of age was not significantly different from that among the age-matched general population (0.38 vs. 0.26; p>0.05; general-population data from Centers for Disease Control and Prevention [CDC], unpub. data). No significant difference was found between the military and general population in the 17- to 29-year-old age group (0.55 vs. 0.40, respectively; p>0.05). The peak disease incidence in military personnel occurred among the 17- to 19-year-old age group and was significantly higher than that in their general-population counterparts (2.34 vs. 0.62; p<0.05; general population data from CDC, unpub. data). This difference was not present in older age categories; incidences among both military personnel and civilians decreased with age.

In succeeding age ranges (20–24, 25–29, and 35–39 years and ages 17–39 combined), the incidence of meningococcal disease among the military was comparable to that in the general population (general population data from CDC, unpub. data). No cases occurred in the military in the 34–39 and >40 age ranges.

### Military Branch and Rank

The mean incidence of meningococcal disease for US military branches ranged from 0.12 for the Air Force to 1.35 for the Marine Corps (Table 2). Of the 11 cases occurring in the 17- to 19-year-old age group, 8 were Marine Corps basic trainees, who are usually 18–21 years of age. Incidence for the Marine Corps was significantly higher than that of the other military branches (p<0.05).

Basic trainees as a group had an incidence of 1.18, significantly higher than enlisted and officer personnel (p<0.05; Table 2). Service branch and rank, however, are confounded. Marine Corps basic trainees represented 35% (9/26) of the total military cases but only 2% of the military population.

### Geotemporal Clustering

While ≈1 million active-duty military personnel (including all recruits) serve in the United States and an additional 400,000 serve around the world, no geographic clustering of cases was observed. In addition, no significant temporal clustering or seasonality of cases was observed.

### Serogroup and Vaccination Status

**Figure 2:**
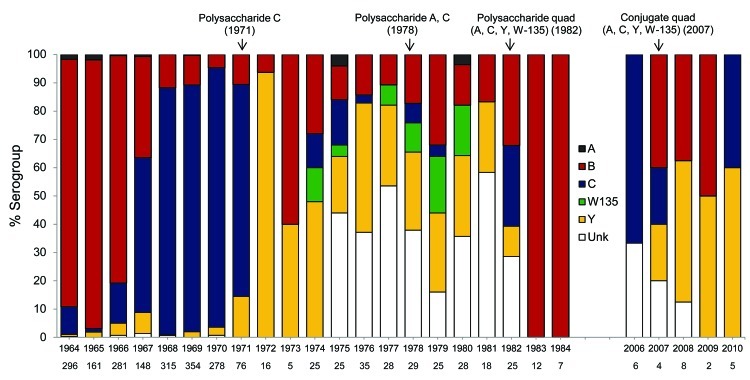
Proportion of each meningococcal serogroup among all isolates tested (1964–1984) or all cases (2006–2010), United States. Years of introduction of vaccine types are indicated by arrows. Unknown during 1964–1980 indicates isolates from a serogroup other than A/B/C/W135/Y or an unknown serogroup; during 1981–1984 indicates isolates that were not B, C, or Y; and during 2006–2010 indicates that no specimen is available and group is unknown. No data were available for 1985–2005. Data for 1964–1984 are from Brundage et al. (*1*) and Brundage and Zollinger (*2*). C, *Neisseria meningitidis* serogroup C; A, *N. meningitidis* serogroup A; quad, quadravalent (*N. meningitidis* serogroup s A, C, W-135, and Y).

The historical and recent proportions of *N. meningitidis* serogroups per year are shown in Figure 2. Among the 26 cases that occurred during 2006–2010, *N. meningitidis* serogroup was determined for 22; 6 (27%) were B, 7 (32%) C, and 9 (41%) Y (Figure 2). In contrast, in the general population during 2006–2009, 24% were B, 45% C, and 28% Y (CDC, unpub. data). Serogroup B was not evident after 2008, and serogroups A and W-135 did not appear during 2006–2010.

Fifteen of the meningococcal disease cases (6 serogroup B) that occurred during 2006–2010 were in persons who had been vaccinated. The mean time from vaccination to illness was 77 months (SD 97) for serogroup B infection. When the polysaccharide vaccine was used, the mean time to illness was 12 months (SD 14) for serogroups C and Y combined; when the conjugate vaccine was used, it was 4 months (SD 2). The apparent difference in mean time to illness for the 2 vaccine groups may be misleading because among the group of 8 persons who received the polysaccharide vaccine was 1 whose time to illness was 43 months.

Seven infections were serogroup C and 8 were serogroup Y; all of these cases occurred in persons vaccinated >21 days before onset of disease. Of those 15 cases, 13 occurred <1 year after vaccination, one <2 years after vaccination, and one 3–4 years after vaccination. Of the 13 cases in which the time between patient vaccination and illness was <1 year, 6 patients had been vaccinated with MPSV-4 and 7 with MCV-4.

Of the 8 cases during 2006–2007 for which the *N. meningitidis* serogroup was determined, 1 was caused by serogroup Y. During 2008–2010, this serogroup was associated with 8 of the 14 cases with known serogroup, 4 of which occurred in persons who had been previously vaccinated with MPSV-4. Nevertheless, this did not represent a significant increase in the prevalence of serogroup Y infection (p = 0.052).

### Fatal Cases

The overall case-fatality rate (CFR) among military personnel with meningococcal disease during 2006–2010 was 19% (5/26 cases), which was not significantly different from the CFR of 13% among the age-matched general population (exact p = 0.24; general population data from CDC, unpub. data). Of the 5 fatal cases, 4 were in persons who had been vaccinated with MPSV-4 (4 deaths among 12 known MPSV-4–vaccinated persons) and 1 in a person vaccinated with MCV-4 (1 death among 12 MCV-4–vaccinated persons).

The Army, Air Force, Navy, and Marine Corps had 3, 1, 1, and 0 fatal meningcococcal disease cases, respectively. Along with a review of data made available by the AFHSC (A. Eick, pers. comm.) and the US Department of Defense Mortality Surveillance Division (L. Pearse, pers. comm.), a review of Army data from various sources published before the current surveillance program began gave us a reliable count of fatal cases during 1997–2005. This, with the Army data cited above (*1*,*2*), enabled us to compare CFRs between historical periods. During 1964–1981, which was the last year of recording until 1997, the CFR in the Army was 6.9%. During 1997–2010, the CFR was 16%. This difference was not significant (p = 0.14).

## Discussion

Although there have been vaccine failures (disease occurring 1–48 months after vaccination), universal vaccination continues to have a protective effect against meningococcal disease in the US military. Analyses indicate that during 2006–2010, even as incidence in the general population was declining, *N. meningitidis* serogroup, clinical syndrome, mortality rates, and overall disease incidence among members of the military were not statistically different from those for the US general population except in the 17- to 19-year-old age group, for which the military rate was higher.

A composite of 100 years of meningococcal disease incidence data for the US Army and general population demonstrates the stark difference in incidences until 1971, when the first polysaccharide *N. meningitidis* serogroup C vaccine was introduced into the military (Figure 1). The introduction of the serogroup A/C vaccine in 1978 further reduced the number of infections caused by 2 of the 3 most prevalent *N. meningitidis* serogroups (the third being serogroup B). From 1990 on, <0.60 cases per 100,000 person-years have been reported. Vaccination against serogroup C may be seen as particularly critical given the history of outbreaks associated with serogroup C in the general population (*6*). Possible reasons for the drop in cases since 1990 are changes in hygiene policies and an increase in routine use of benzathine G penicillin as prophylaxis against group A streptococcal infections in military recruit camps starting in 1991 (J. Brundage, pers. comm.). Previous studies have demonstrated a relationship between meningococcal infection rates and respiratory disease rates, which have also fallen in the military population over the same period, although the association is not entirely consistent (*9*–*12*).

During 2000–2010, the differences in overall incidence between the Army, the military as a whole, and the general US population were small (incidences of 0.40, 0.49, and 0.44, respectively). Although lower incidence may be expected within the universally vaccinated military population, several factors put military members at higher risk, such as a high percentage of relatively young personnel originating from disparate regions and populations with differing carriage rates, as well as challenging operating environments and crowded living conditions. The risk is evident in the military’s elevated rates of other infectious diseases, such as respiratory infections (*9*–*12*); however, Artenstein et al. (*13*) suggested that elevated respiratory disease rates were not likely to be relevant. The current infection rates among the military may represent a limit of vaccine effectiveness in this highly vulnerable population.

The year-to-year proportion of *N. meningitidis* serogroups among meningococcal infections has varied in the US general population (review of 2002–2009 annual Active Bacterial Core Surveillance reports; *5*) and the military population (Figure 2). As expected, the percentage of serogroup C infections was reduced dramatically after the introduction of the serogroup C vaccine, and consequently, so was the total number of meningococcal disease cases. Data from 1981–2005 are not available, but it appears that the quadrivalent polysaccharide vaccine was successful in reducing infection rates for all serogroups even further, perhaps by as much as 80% (Figure 1, Figure 2).

During 2007–2008, the military gradually switched from the polysaccharide vaccine (MPSV-4) to the conjugate vaccine (MCV-4). For the cases in our study from 2006–2007 for which vaccine information was available, all but 1 person was vaccinated with the MPSV-4. A spike in cases occurred in 2008, the first year that MCV-4 was universally adopted, but only half of those infected had been vaccinated with MCV-4. It is too soon to determine whether the change to MCV-4 has had an effect on the overall incidence of meningococcal disease in the military population.

Of the 15 vaccine failures (disease occurrence 1–48 months after vaccination), 12 occurred <12 months after vaccination and 2 occurred 1–4 years after vaccination. The unique conditions of the first year of the military-training environment may lead to relatively high rates of observed vaccine failures. The number of recruits entering the military each year has been relatively constant; thus, so has the number of vaccinations. No trend was seen for vaccine failures (4, 1, 3, 1, 4 for the years 2006–2010, respectively), and no data suggest a different rate of vaccine failure for the 2 vaccines. The expectation with the newer MCV-4 was that disease incidence would diminish, but so far, that does not appear to be the case.

Although studies have demonstrated the waning of correlates of protection over time after initial meningococcal vaccination (*14*,*15*), most military personnel do not receive a booster during their military careers (*4*). Boosters are effective (*16*) and are given 5 years after vaccination to personnel traveling to high-risk areas. A study to gather serologic data to address the question of long-term vaccination coverage is underway.

During 2006–2010, *N. meningitidis* serogroup distribution in the military was similar to that for the age-matched general population (general population data from CDC, unpub. data), although the military has seen more serogroup Y infections during the past 3 years when compared with the historical predominance of serogroup C. Serogroup distribution varies year-to-year in the general population; a large increase in the percentage of serogroup Y infections was seen during 1989–1991 (*17*). The increase in serogroup Y infections in military personnel is contemporaneous with the changeover to the MCV-4 vaccine, but half of those cases were in persons who had been vaccinated with MPSV-4. Our data cover only the 2–3 years after the adoption of MCV-4 in the military and are insufficient to support claims about the effect of vaccine change on serogroup Y incidence; thus, we were unable to identify any vaccine-related trends in cases related to particular serogroups. All of the serogroup Y infections have occurred in persons <25 years of age (Table 2).

*N. meningitidis* serogroup B is not included in the vaccines, but this absence has not led to its dominance over other circulating strains that are included in the vaccine. In fact, serogroup B accounts for fewer infections than serogroups C and Y, which suggests an unknown factor is preventing an increase in the proportion of serogroup B infections in the military.

As in the general US population, the highest incidence of meningococcal disease in the military was among young adults, but the incidence among 17- to 19-year-old military personnel exceeded that among the same age group in the general population (general-population rate from CDC, unpub. data). However, rates for the military should be compared with those in similar general settings, such as college dormitories, where rates as high as 5.1 have been reported (*18*).

In the Army, the 1997–2010 CFR of 16% for meningococcal disease appears strikingly higher than the CFR of 7% for 1964–1981, a period spanning the use of 3 different vaccines (*1*). Given that the Army had only 1, nonfatal case during 2008–2010, it is reasonable to suggest that the high CFR during 1997–2010 is an anomaly.

In this study, while constituting only 14% of the total military population, the Marine Corps accounted for half the total cases of meningococcal disease in the military. In particular, Marine Corps recruits contributed a disproportionate 7 of 11 cases among the 17- to 19-year-old age group. Given that most cases across all military branches were among basic trainees (mainly the 17- to 19-year-old age group), among whom the rate of 1.18 is significantly higher than for enlisted and officer personnel, and that most of the basic trainee cases were from the Marine Corps, the exceptional rate of meningococcal disease in the Marine Corps is thus a function of the Marine Corps basic training rate. One possible reason for the high rate of infection among Marine Corps basic trainees is that the basic training period is 4 weeks longer than that of other branches, although only 2 of 8 Marine Corps cases occurred during the last 4 weeks of training. In addition, data from Brundage et al. (*1*) and AFHSC (Angelia Eick, pers. comm.) show that the Marine Corps rates over previous 5- and 10-year periods were 0.34 and 0.47, respectively. Thus, the current 5-year rate may be a short-term anomaly.

In summary, while meningococcal disease has historically been associated with substantial illness among members of the US military, disease incidence during the past 5 years is at a historic low and is comparable to the rate among the age-adjusted US population. The current rates in the military have been maintained since the 1990s and can be attributed to effective meningococcal vaccines. While some observed trends suggest covariation between length of basic training, age, and service branch, surveillance has not shown consistent trends by which military rates meaningfully differ from those in the general population. Covariation was seen, however, between the higher rate among basic trainees, age group, and Marine Corps versus the rest of the military.

In the military setting, and especially in the basic training setting, epidemic meningococcal disease remains controlled due to a robust immunization program. To date, no evidence of a change in overall disease rates has been associated with the switch to MCV-4 vaccine. However, the increase of serogroup Y infections among the military population warrants further observation.
